# Virtual vitreoretinal clinics: a service delivery pathway of the future

**DOI:** 10.1186/s40942-025-00684-3

**Published:** 2025-06-01

**Authors:** Elizabeth Yang, Amelia Rees, Shantelle Ahadzi, Yvonne Kanna, Philipp Schwember, Robert Henderson, Lyndon da Cruz

**Affiliations:** 1 Moorfields Eye Hospital, 162 City Road, London, EC1V 2PD UK; 2 University of Manchester, Oxford Rd, Manchester, M13 9PL UK; 3 Sahlgrenska University Hospital, Göteborg, 413 45 Sweden

**Keywords:** Vitreo-retinal surgery, Virtual clinics, Virtual pathways, Retinal imaging, OCT, Service delivery

## Abstract

**Background:**

Vitreo-macular interface (VMI) disorders, including epiretinal membrane (ERM) diagnosed on optical coherence tomography (OCT), form a significant proportion of elective referrals to vitreoretinal (VR) surgeons. An in-person visit to a clinician involves travelling, waiting, investigations then an interaction with the surgeon, which entails many inefficiencies in a large institution. We report the pilot studies of a VR virtual service where these patients can be more efficiently reviewed, investigated, listed for surgery or discharged.

**Methods:**

This was a prospective observational study comparing the outcomes of a virtual assessment to standard face-to-face clinics. All patients included were referred from optometry practices for ERM diagnosed on macula OCT. A first pilot study comprised 79 patients, who attended a diagnostics centre staffed with ophthalmic-trained technicians. A short history, visual acuity and ocular pressures were recorded. Widefield colour photographs and macular OCT images were acquired. Cases were asynchronously reviewed by trained ophthalmologists and senior nurses within the week, and following a telephone consultation with the patient, a virtual management plan was documented. All patients attended 1 week later for a face-to-face appointment, following which, virtual and face-to-face management plans were compared. A second pilot comprised 65 patients, through the same pathway, to examine consistency. A post-hoc analysis was carried out to identify the cohort of patients who would be suitable for a virtual management decision without a telephone consultation.

**Results:**

ERMs comprised 35% of overall elective referrals in this study. In Pilot 1, 42% were virtually assessed for discharge, with high concordance with face-to-face outcomes (positive predictive value = 89%). There were 3 cases of missed retinal tears, and 1 OCT misdiagnosis. In the second pilot, 43% were discharged virtually, with higher concordant discharge rates (positive predictive value = 93%). There were no missed peripheral pathology and no misdiagnoses in this pilot.

**Conclusions:**

Our virtual model demonstrates a safe and effective way of managing and discharging patients without a face-to-face clinic. This is especially suitable for low-risk conditions such as ERMs, which comprise a large proportion of referrals.

## Background

Virtual or remote assessments have been effectively utilised in the United Kingdom for several years in ophthalmology, for example to evaluate glaucoma patients [1, 2]. Alongside the screening of diabetic retinopathy through the UK Diabetic Eye Screening Programme, numerous hospitals have also established virtual clinics to monitor patients with stable medical retina conditions, such as treated neovascular age-related macular degeneration and mild non-proliferative diabetic retinopathy [3–5]. Virtual assessment pathways offer a valuable solution for managing an ever-growing patient population, allowing in-person clinic visits to be prioritized for urgent assessments and treatment.

In Moorfields, we have previously published a proof-of-concept study on virtual clinics for post-operative patients with retinal detachments [6], and other units in the UK are looking to incorporate hybrid telemedicine for VR patients [7]. There are otherwise few other studies that have looked at the outcomes of virtual assessments of VR patients as a study cohort.

The Moorfields Eye Hospital VR service has two main referral streams: the VR emergency service that processes urgent conditions such as retinal detachments and trauma, and the elective referral stream. Elective referrals are typically from optometrists, general practitioners or internal referrals from other ophthalmology specialist departments that require a non-urgent VR opinion.

During the COVID-19 national lockdown in the UK, it is estimated that the number of retina patients seen at Moorfields Eye Hospital was less than 30% of normal clinical capacity. In the VR department, the focus was on emergency surgery primarily for trauma and retinal detachments, and on managing high-risk cases in a safe and socially distanced manner [8]. There was subsequently a large increase of new referrals in the aftermath of the pandemic, which coincided with an increased use of OCT imaging in high street optometry practices. This translated to elective referral waiting times of more than 4–6 months for low-risk macula conditions such as ERMs to the VR service.

**Fig. 1:**
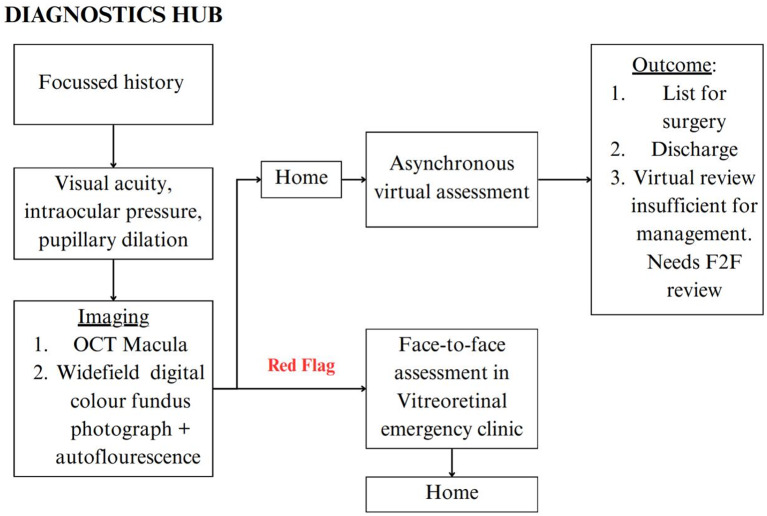
The diagnostics hub VR virtual clinic pilot pathway. New patients referred with ERM attend the diagnostics hub, staffed by imaging technicians. If any red flags (suspected retinal detachment, red and painful eye, IOP < 5 or > 35), technicians contact the VR emergency team at Moorfields City Road for a same day assessment. If there are no red flags, patients are sent home, their imaging and case reviewed asynchronously within 1 week by the virtual assessment clinicians. As per pilot design, all patients attended a subsequent face-to-face (F2F) traditional clinic, so that outcomes could be compared

In our Diagnostic Hub model (Fig. 1) for a virtual VR pathway, initially utilised by the Moorfields Medical Retina service [9], patients visit the Moorfields Eye Hospital Diagnostics Hub for evaluations conducted by ophthalmic-trained technicians. These assessments include history taking, best-corrected visual acuity (BCVA) testing, intraocular pressure (IOP) measurement, and retinal imaging. Clinical parameters are reviewed asynchronously through a virtual assessment, leading to a diagnosis and management plan. This is communicated through a telephone consultation.

In this study, we investigate if this model of virtual consultations could enhance the efficiency of ERM referrals and mangement, as a pilot for all VMI disorders. This could free up more face-to-face clinic consultations for urgent VR conditions such as advanced diabetic eye disease or complicated retinal detachments for example, and improve our patient flow in a targeted manner.

## Methods

This was a prospective observational study, completed following institutional board approval by the Audit Department of Moorfields Eye Hospital NHS Foundation Trust. This study adheres to the tenets set forth in the Declaration of Helsinki.

### Study cohort

All consecutive referrals to the Moorfields VR service with a query of ERM were included. The Pilot 1 cohort comprised all ERM referrals in the month of November 2022 (*n* = 79), and the Pilot 2 cohort included all ERM referrals in the month of October 2023 (*n* = 65). Patients were excluded if they did not complete both the virtual assessment and the subsequent face-to-face review, or if they were already under follow-up and not new referrals.

### Virtual pathway

**Fig. 2:**
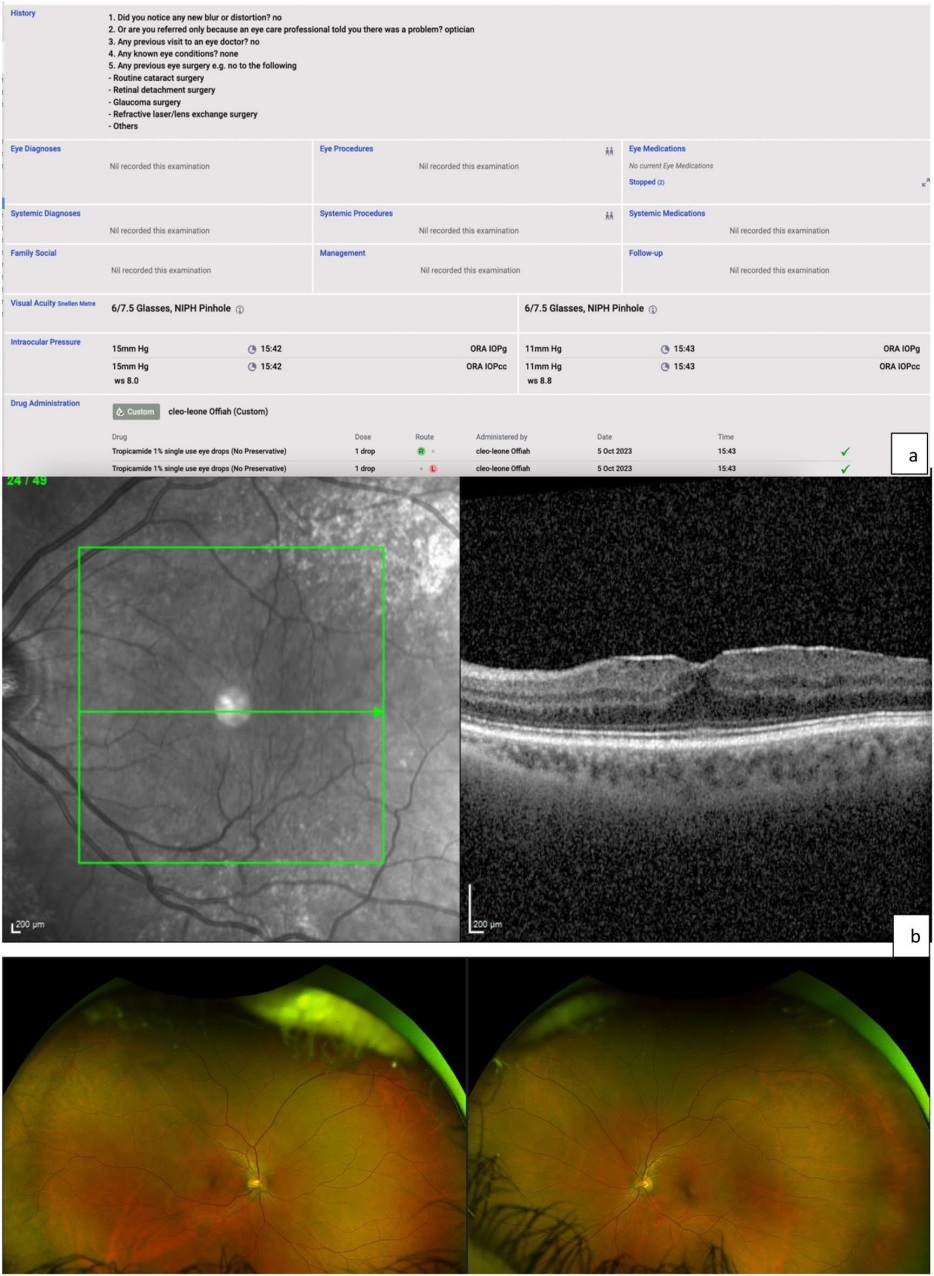
(**a**) Technician’s data input on the OpenEyes electronic records system, with 5 triage questions, BCVA, IOP, and all patients were dilated. (**b**) Example patient with macular OCT imaging (Spectralis, Heidelberg) showing ERM on the left eye. In the virtual diagnostics hub, both eyes are imaged but only the referral eye is shown here. (**c**) Widefield (Optos) imaging of both eyes of an example patient at the virtual diagnostics hub. Both eyes are imaged. Superior and inferior views, as well as autofluorescence images are also taken, which are not shown here

New patients referred for ERM attended the Diagnostics Hub, a stand-alone Moorfields facility staffed by imaging technicians, as per the Diagnostics Hub model (Fig. 1). The technicians recorded a focussed clinical history, BCVA, IOP, acquired macular OCT scans (Heidelberg Spectralis) and widefield digital colour photographs (Optos), with all patients having pupillary dilation (Fig. 2). Patients with denoted “red flag” features (suspected retinal detachment, red and painful eye, IOP < 5 or > 35) were redirected to an urgent face-to-face assessment in the VR emergency clinic.

Virtual reviews were conducted asynchronously during the same week, either by a VR consultant, three VR fellows, or two experienced VR-trained nurse practitioners. The fellows are VR-trained specialist surgeons who are able to run independent VR clinics and lists, and VR nurses are the dedicated nurse practitioners who solely work in VR emergency and outpatient clinics for a minimum of 5 years. Management plans were documented as one of the following: (a) discharge (b) list for surgery or (c) virtual review insufficient for management plan, face-to-face review required. All 79 patients then attended a standard face-to-face clinic the following week, where a different clinician, masked to the virtual outcome, independently assessed the patient. Subsequently, the management plans from the virtual and face-to-face reviews were compared to assess the safety and reproducibility of virtual clinics, with the outcomes from the face-to-face consultation taken as gold standard.

A second cohort of 65 patients referred with ERMs in October 2023 underwent the same virtual pathway, conducted a year later to evaluate the reproducibility of this service. This followed a year of enhanced nurse practitioner training, aimed at improving OCT interpretation and virtual decision-making accuracy.

### Post-hoc analysis

Two VR fellows (EY and PS) independently and retrospectively reviewed the initial optometric referrals, virtual imaging, BCVA and IOP from the Pilot 2 cohort. They determined which patients were (A) suitable for discharge without telephone consultation, or (B) not suitable for virtual review alone and required either a telephone call or in-person clinic assessment. Both reviewers were masked to the standard face-to-face outcomes. Inter-observer agreement and concordance with the face-to-face decision were analysed.

### Statistical analysis

All data were analysed descriptively using both Microsoft Excel (Microsoft Corp.) and Python (version 3.9.22) (Van Rossum, G., & Drake, F. L. (2009). *Python 3 Reference Manual*. Scotts Valley, CA: CreateSpace). Diagnostic accuracy metrics including positive predictive value, sensitivity, and specificity were calculated in order to compare the clinical outcomes from virtual assessments with face-to-face clinical outcomes, which were taken as the reference standard.

Cohen’s Kappa coefficient was also calculated to assess inter-method agreement between virtual and face-to-face management plans for discharge and listing for surgery, beyond what would be expected by chance (Cohen, J. (1960). These analyses were performed using Python’s *scikit-lean* and *pandas* libraries. No formal hypothesis testing was conducted, as this was a descriptive and exploratory pilot study.

## Results

### Pilot 1

ERMs comprised 35% of all in elective referrals in this time period. 79 referral diagnoses of ERM underwent virtual review and telephone consultation.

**Fig. 3:**
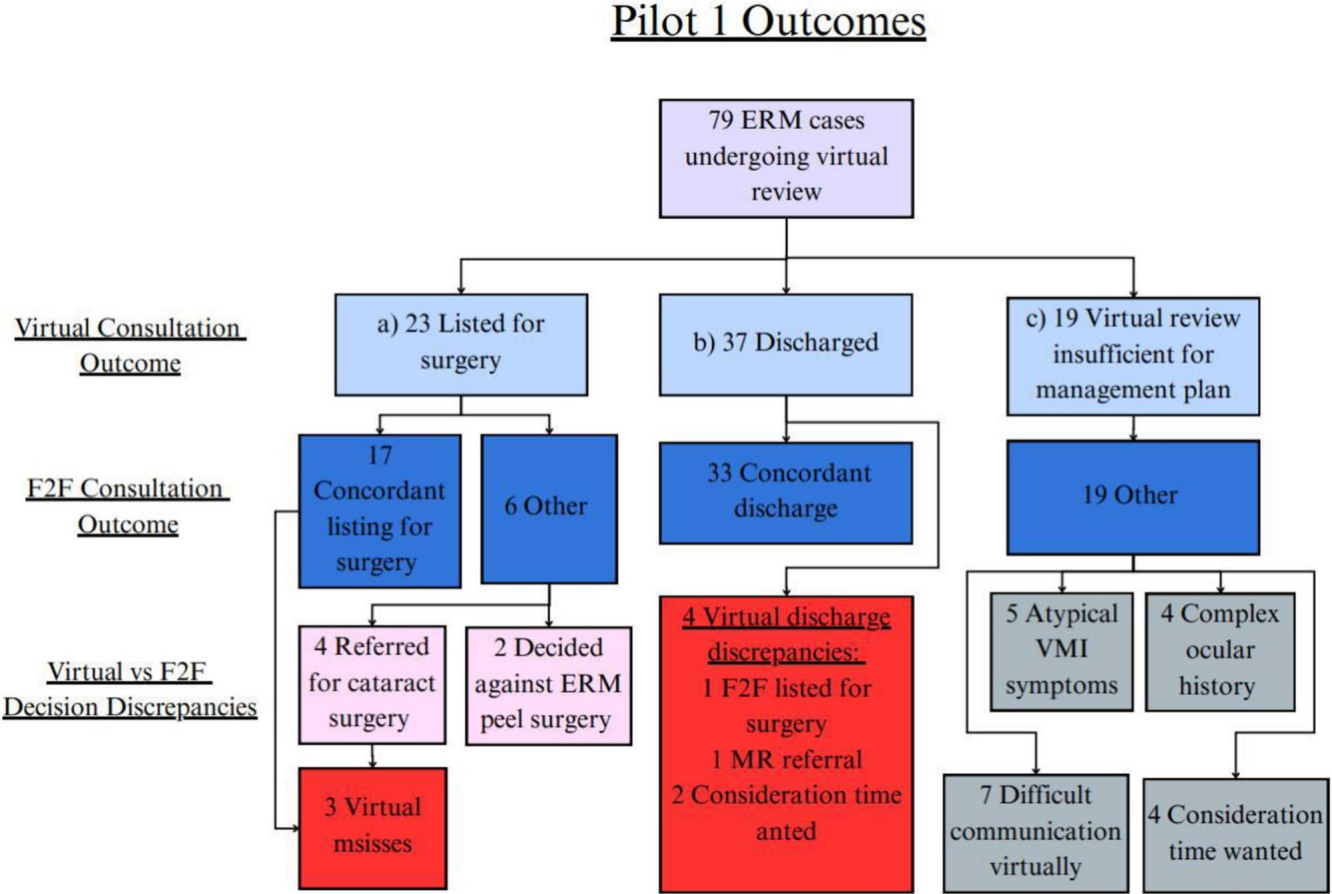
Flowchart of Pilot 1 ERM patients and virtual outcomes, which are **a**) list for surgery, **b**) discharge, or **c**) virtual review insufficient, needs face-to-face clinic for decision. These are compared with the masked face-to-face (F2F) clinic outcomes. Decision discrepancies are noted, as well as reasons for virtual review being insufficient for making a management plan. Details are elaborated in the manuscript text

For all virtual reviews, management plan options were either (a) list for surgery, (b) discharge, or (c) virtual review insufficient, needs face-to-face clinic for decision (Fig. 3).

### Listing for surgery

Of the 79 patients, 23 (29%) were listed for surgery based on the virtual consultation. Of these, 17 patients (22%) were also listed for surgery at the face-to-face clinic, resulting in a positive predictive value of 74%. The sensitivity and specificity of virtual assessments for surgery listing were 74% and 89% respectively (Table 1).

### Discharge

37 (47%) were discharged as the plan from the virtual consultation, while 44 (56%) were discharged following the face-to-face clinic (positive predictive value = 89%). Among these, 33 patients (42%) were discharged in agreement by both virtual and face-to-face assessments. This extrapolates to a concordant discharge rate of 21.4% for all elective new referrals in this pilot. The sensitivity and specificity of virtual discharge decisions were 75% and 89% respectively (Table 1).

Agreement between virtual and face-to-face management plans in Pilot 1 was further assessed using Cohen’s Kappa coefficient, which showed moderate agreement for both discharge decisions (κ = 0.63) and surgery listing decisions (κ = 0.60).

**Table 1:** Pilot 1 agreement between virtual decisions and masked face-to-face clinic decisions. Sensitivity and specificity of virtual decisions for listing for surgery and discharge are noted

Management Plan	True Positives (Agreement)	False Positive (Disagreement)	Positive Predictive Value	Sensitivity (%)	Specificity (%)	Total
Listed for Surgery	17 (73.9%)	6 (26.1%)	73.9%	73.9%	89.3%	23
Discharged	33 (89.2%)	4 (10.8%)	89.2%	75.0%	88.6%	37

### Virtual discharge discrepancies

Of the patients discharged virtually in Pilot 1, there were four cases where the face-to-face management plan was in disagreement. One patient, reviewed virtually via telephone with a nurse practitioner and discharged, was later listed for vitrectomy with ERM peel after in-person consultation with a consultant. This patient had diabetic macular oedema with a concurrent ERM. A second patient, also discharged virtually following a nurse practitioner consultation, was referred for a medical retina opinion at the face-to-face clinic due to concerns of macular telangiectasia. The remaining two patients wanted some time to consider ERM peel surgery and a further appointment was made.

### Virtual review insufficient for management plan

19 patients (22%) could not receive a definitive management plan through virtual review alone and were therefore required to attend for face-to-face assessment. Of these, five had poor-quality imaging due to media opacity, and their symptoms did not correspond to a vitreo-macular interface disorder. Seven patients experienced communication difficulties during the telephone consultation the phone. Four had complicated ocular histories, including previous glaucoma surgery or proliferative diabetic retinopathy, requiring additional imaging and in-person evaluation. The remaining four patients requested additional time to consider surgery before making a decision.

### Virtual misses

Three patients were found to have retinal tears that were only detected during their mandatory face-to-face clinic visit. Two of these patients had been concordantly listed for vitrectomy and ERM peel, while one had been referred for face-to-face assessment to discuss cataract surgery. Of the three, two had been reviewed virtually by nurse practitioners, and one by a consultant. All three underwent successful laser retinopexy following detection of the tears in person.

As mentioned above, one patient listed virtually for ERM peel by a nurse practitioner was subsequently referred to the medical retina service during the face-to-face visit and diagnosed with macular telangiectasia.

### Pilot 2

**Fig. 4:**
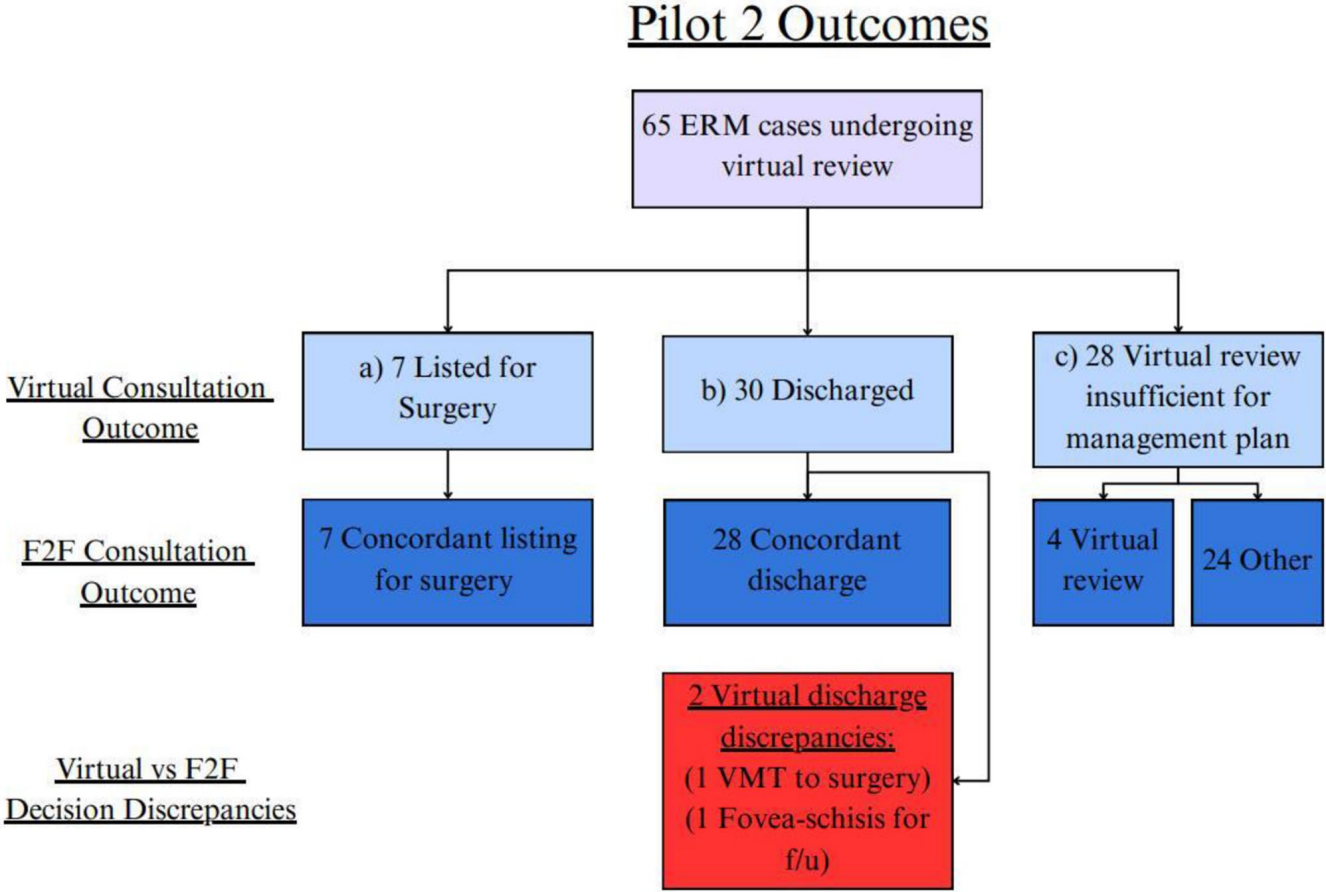
Flowchart of Pilot 2 ERM patients and virtual outcomes, which are **a**) list for surgery, **b**) discharge, or **c**) virtual review insufficient for management plan. These are compared with the masked face-to-face (F2F) clinic outcomes. Decision discrepancies are noted, as well as reasons for virtual review being insufficient for making a management plan. Details are elaborated in the manuscript text. f/u: follow up

Pilot 2 comprised 65 patients referred with ERMs, and was conducted one year after Pilot 1 using the same virtual assessment pathway (Fig. 4).

### Listing for surgery

7 patients (10.8%) were listed for surgery at virtual review, all of which were confirmed for surgery at their face-to-face clinic (positive predictive value = 100%). The sensitivity and specificity of virtual assessments for surgery listing were both 100% (Table 2).

### Discharge

28 (43.1%) were discharged following virtual consultation, and all were concordantly discharged at the face-to-face clinic the following week (positive predictive value = 93.3%) (Table 2). Cohen’s Kappa analysis demonstrated substantial agreement for discharge decisions (κ = 0.63) and perfect agreement for surgery listing decisions (κ = 1.00).

**Table 2:** Pilot 2 agreement between virtual decisions and masked face-to-face clinic decisions. Positive predictive value, sensitivity and specificity of virtual decisions for listing for surgery and discharge are noted

Management Plan	True Positives (Agreement)	False Positive (Disagreement)	Positive Predictive Value	Sensitivity (%)	Specificity (%)	Total
**Listed for Surgery**	7 (100.0%)	0 (0.0%)	100.0%	100.0%	100.0%	7
**Discharged**	28 (98.3%)	2 (6.7%)	93.3%	65.1%	90.9%	30

### Virtual discharge discrepancies

There were two cases of discrepancies in discharge decisions in the Pilot 2 study. One patient with vitreo-macular traction and good visual acuity was discharged virtually, but was listed for surgery following in-person consultant assessment due to symptoms being deemed significant. The second patient, with mild ERM and asymptomatic myopic foveoschisis, was discharged following virtual review, but was scheduled for a 6-month review at their face-to-face visit with the consultant.

### Virtual review insufficient for management plan

24 patients (37%) in did not receive a definitive management plan during the virtual review and therefore were brought in for a face-to-face assessment. Of these, 10 patients (42%) had language barriers affecting the telephone consultation, 6 (25%) had insufficient imaging quality, 6 (25%) required more time to consider surgery, and 2 (8%) raised concerns of other potential pathology.

4 patients had an accurate diagnosis at virtual review (2 fovea-schisis, 2 VMT) but needed routine follow up for monitoring, in agreement with the face-to-face decision.

### Virtual misses

There were no cases of missed pathology such as retinal tears or erroneously diagnosed ERMs in Pilot 2. There was a single case of a virtual identification of a temporal retinal haemorrhage suspicious for a retinal tear on Optos widefield imaging, which was later confirmed as a retinal tear at the face-to-face clinic and successfully treated with laser retinopexy.

## Other findings

### Patient appointment waiting times

In both pilot cohorts, patients spent an average of 39 min in the virtual diagnostics hub, followed by an average of 16 min for the asynchronous virtual consultation, typically conducted in their own homes. In contrast, patients attending the face-to-face VR clinics, spent an average of 85 min in the hospital per appointment.

### Post-hoc analysis

In the post-hoc triage review of all referrals, we analysed retrospective decisions made by either or both fellows for (A) suitable for discharge without telephone consultation, or for (B) patients for which virtual review alone is insufficient.

Both fellows agreed with 100% concordance (Cohen’s Kappa analysis [κ = 1.00]) in identifying the patients suitable for discharge without telephone consultation, for which all of these patients were also discharged on the gold standard face-to-face management plan. This cohort of patients represent those who have good vision, with no documented visual disturbance, no other complicating ocular pathology, and with minimal anatomical distortion on macular imaging, therefore unlikely to require any intervention. Both fellows also agreed with 100% concordance with Cohen’s Kappa analysis (κ = 1.00) for patients for which virtual review alone is insufficient.

## Discussion

The prevalence of idiopathic ERMs increases with age, affecting up to 34% of the population over 60 years of age [10]. Unsurprisingly, ERMs form 35% of all elective referrals to Moorfields, representing a significant proportion of elective clinics. While this data for other units in the UK is currently limited, this proportion in Moorfields has increased over the last 5 years (unpublished local data), which is in keeping with the increased availability of OCT imaging in local optometry practices.

The increasing workload therefore prompted us to devise more efficient methods of triaging patients, starting with ERMs as the single most common referral condition.

In current practice of virtual clinics in ophthalmology, the most notable risk of that is safety and missed pathology. Current published models demonstrate that the risk can be reduced by high quality imaging and experienced reviewers [11, 12]. We tested our virtual assessment model through two pilots, the second refined from the first, to identify the safety and repeatability of this model.

### Pilot 1

The first pilot was a feasibility study of a new virtual pathway through the diagnostics hub. Clinicians acted cautiously in the virtual reviews and only made clear discharge decisions when patients were extremely low risk. As a result, the discharge rate virtually was lower (47%) than the face-to-face discharge rate (56%), with a concordant discharge rate of 42%.

In this pilot, we wanted to test the safety of a virtual review. Notably, there were 4 patients who were virtually decided for discharge, but in the subsequent face-to-face clinic, 1 was listed for surgery, 1 referred to medical retina, and 2 wanted time to re-consider a vitrectomy/ERM peel.

The patient that was listed for surgery face-to-face was a complex case with treated proliferative diabetic retinopathy, with macular oedema and a concurrent ERM and macular ischaemia. In retrospect, complex cases are not well-suited for virtual review with non-consultant nurse practitioners.

The patient who was referred to medical retina by a VR fellow in the face-to-face clinic had features of macular telangiectasia type 2. This patient had been reviewed virtually by a nurse practitioner, and the decision was for discharge as they had very good vision, no symptoms of distortion, but they did not recognise the macular telangiectasia features.

Following this pilot, our nurses underwent intensive OCT interpretation exercises with the VR fellows and participated in regular clinics over the year, to improve upon macular imaging interpretation. This was successful as shown in Pilot 2, with no errors in diagnoses.

With regards to peripheral pathology, it is worth noting that in Pilot 1 there were 3 cases of missed peripheral retinal breaks.

**Fig. 5:**
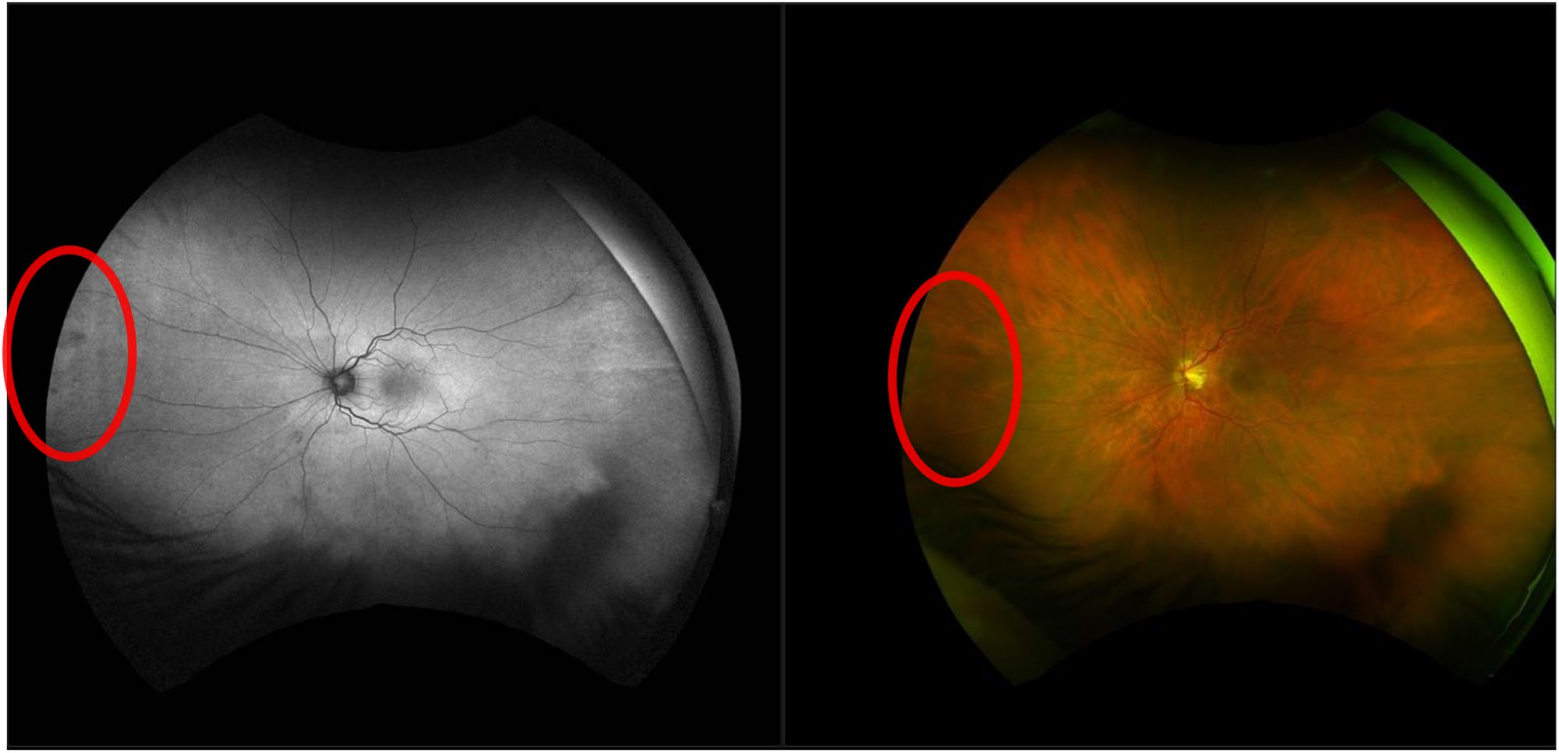
Example of a subtle left nasal retinal tear that was not detected on virtual review. This was noticed in clinic, and treated with laser retinopexy on the same day

At the virtual review of these 3 missed tears, 1 was by a consultant, 1 by a fellow and 1 by a nurse, suggesting that clinical seniority is less relevant than thorough imaging review. All 3 retinal breaks were present on the virtual Optos image (Fig. 5) but were subtle. Reassuringly, as a testament to the safety of our pilot design, all virtual patients were mandated to return for a face-to-face clinic, at which all 3 breaks were diagnosed and successfully treated.

### Pilot 2

Pilot 2 was a replicate of Pilot 1 but after a year of nurse practitioner training on OCT imaging and ERM management. This allows us to examine most common routine referral diagnosis (35%) with greater clarity and focus. Learning from the missed peripheral pathology in Pilot 1, we enforced a methodical step-wise approach to widefield imaging review. We reviewed the colour images starting at the posterior pole, followed up all visible periphery starting in a clockwise manner from 12 o’clock, in a magnified view, followed by the same sequence in the autofluorescence images.

In this pilot, there were no further missed peripheral pathology, and no other misdiagnosed macular conditions. There was a single case of a virtually detected retinal tear, which was confirmed and treated in face-to-face clinic. The 0% missed pathology this pilot is likely thanks to a year-long nurse practitioner training on OCT imaging and macular disorders, and methodical peripheral widefield image evaluation.

There were 2 discrepancies in discharge decisions in this pilot, one was a patient who changed his mind about surgery when attending face-to-face, and the other was a myopic fovea-schisis who was decided in the face-to-face clinic for a 6-month review instead of a discharge. These discrepancies reflect clinician variability rather than any missed pathology on the virtual review.

In both pilots, there was a proportion of patients (24% in Pilot 1, 37% in Pilot 2) where virtual consultation was not suitable, common reasons being a language barrier making telephone consultations to clarify symptoms challenging, and hazy media with sub-optimal quality imaging, and difficulty in differentiating between cataract symptoms or VMI symptoms. Unsurprisingly, digital exclusion represents a common theme in all current virtual consultation models in medicine [13, 14].

As we move forward in implementing the VR virtual service, we are considering video consultations with booked interpreters or family members to reduce digital exclusion of non-English speakers.

### Post-hoc analysis

Reducing telephone consultations can reduce the clinician time spent, increase capacity, and reduce the staffing required for the virtual pathway.

In the post-hoc analysis, we identified patients who were deemed low risk, suitable for discharge with a letter, without a telephone consultation. Independently, they agreed that these were patients who had good BCVA better or equal to 6/9, no previous ocular history of surgery, no visual complaints, and with anatomically minimal ERM on imaging.

Both observers also agreed on referrals that were not suitable for virtual review, for example patients with e.g. advanced retinal disease, complicated ocular history, and media opacity, who more benefit more from face-to-face examination and discussion. Recognising referrals unsuitable for a virtual pathway can similarly optimise patient flow.

### Advantages of the VR virtual clinic model

A major advantage of our virtual clinic model is convenience for patients. Overall, the patients were seen in the diagnostics hub without any delay, and the average time spent in the hub was much shorter than our regular face-to-face clinics, where patients are often seen more than 2 h later than their planned appointment times. They also had the flexibility of a virtual consultation with clinicians from their own homes.

Another advantage of virtual clinics is that of clinical efficiency. We show that we can concordantly and safely discharge 42% and 43% for Pilot 1 and 2 respectively. Along with the proposed changes in appropriate risk-triage and reducing telephone calls, and improved clinicians experience in virtual consultations, there is a potential to expand the virtual capacity which allows us to address larger patient volumes in the same amount of time as a traditional clinic. This will lead to reduced waiting times from referrals to appointments.

The pilots additionally showed that remote listing for surgery is potentially feasible. Although not tested here, our future virtual pathway could trial direct listing for surgery, possibly requiring additional imaging to assess the need for combined cataract and vitrectomy procedures.

### Disadvantages of the VR virtual clinic model

A potential concern of virtual pathways is that of safety. The purpose of running two pilots, one building on the other, was to minimise risk to patients. The pilots were useful in assessing the safety of imaging reviews, and although there were cases of missed pathology in Pilot 1, with further clinician training and experience in methodical virtual imaging reviews, there were no cases of missed pathology in Pilot 2.

We also involved nurse practitioners from the start of this project, to increase staffing and clinic capacity, in order to successfully lower the waiting times for routine referrals. By the second pilot, nurse practitioners were equal to consultants and fellows in successful virtual reviews for VMI disorders, which was reassuring. In the future, there is scope to expand our virtual hubs into the community, with direct links to optometry and primary care, to further improve on referral efficiency.

## Conclusion

These pilots have shown that our model of virtual clinics in VR is feasible, can improve efficiency and patient waiting times to a clinical review. We have shown that this model is a safe way of managing routine ERM referrals, and patients who are suitable for a virtual review can be identified. Patients also spend little time in the diagnostics hub, and have highly rated the virtual process. In the future, we will pilot further categories of pathology that can be safely managed virtually, train more nurse practitioners to expand our capacity, and work with primary care referrers to fine tune the quality of referrals.

## Data Availability

No datasets were generated or analysed during the current study.

## Abbreviations

VR: Vitreo-retinal
VMI: Vitreo-macula interface
OCT: Optical coherence tomography
ERM: Epi-retinal membrane
F2F: Face-to-face
f/u: Follow up

## References

[1] Kotecha A, Brookes J, Foster PJ. A technician-delivered ‘virtual clinic’ for triaging low-risk glaucoma referrals. Eye (Lond). 2017;31:899–905. 10.1038/eye.2017.9. [DOI] [PMC free article] [PubMed] [Google Scholar]. 28211881 10.1038/eye.2017.9 PMC5518844

[2] Ratnarajan G, Kean J, French K, Parker M, Bourne R. The false negative rate and the role for virtual review in a nationally evaluated glaucoma referral refinement scheme. Ophthalmic Physiol Opt. 2015;35:577–81. [DOI] [PubMed] [Google Scholar]. 26088949 10.1111/opo.12224

[3] Scanlon PH. The english National screening programme for diabetic retinopathy 2003–016. Acta Diabetol. 2017;54:515–25. 10.1007/s00592-017-0974-1. [DOI] [PMC free article] [PubMed] [Google Scholar]. 28224275 10.1007/s00592-017-0974-1 PMC5429356

[4] Tsaousis KT, Empeslidis T, Konidaris VE, Kapoor B, Deane J. The concept of virtual clinics in monitoring patients with age-related macular degeneration. Acta Ophthalmol. 2016;94:e353–355. [DOI] [PubMed] [Google Scholar]. 26385270 10.1111/aos.12832

[5] Kern C, Kortuem K, Hamilton R, Fasolo S, Cai Y, Balaskas K, et al. Clinical outcomes of a hospital-based teleophthalmology service: what happens to patients in a virtual clinic? Ophthalmol Retin. 2019;3:422–8. [DOI] [PubMed] [Google Scholar]. 10.1016/j.oret.2019.01.011 31044734

[6] Anguita R, Ahmed S, Makuloluwa A, Hind J, Roth J, Wickham L. Prospective validation of a virtual post-operative clinic in vitreoretinal surgery. Eye (Lond). 2024;38(17):3258–62. 10.1038/s41433-024-03272-1. Epub 2024 Jul 26. PMID: 39060343; PMCID: PMC11584701. 39060343 10.1038/s41433-024-03272-1 PMC11584701

[7] Makanjuola T, Schneiders M, Chawla A, Wilson K, Spiteri Cornish K. Telemedicine: outcomes of a hybrid vitreoretinal service, from pilot to practice. Eye (Lond). 2024;38(6):1221–2. 10.1038/s41433-023-02866-5. Epub 2023 Dec 1. PMID: 38040963; PMCID: PMC11009273. 38040963 10.1038/s41433-023-02866-5 PMC11009273

[8] Royal college of ophthalmologists. Guidance on restarting medical retina services. London, UK: Royal College of Ophthalmologists; 2020.

[9] Hanumunthadu D, Adan K, Tinkler K, Balaskas K, Hamilton R, Nicholson L, Moorfields Medical Retina Virtual Assessment Study Group. Eye (Lond). 2022;36(3):627–33. 10.1038/s41433-021-01510-4. Epub 2021 Apr 6. PMID: 33824508; PMCID: PMC8023775. Outcomes following implementation of a high-volume medical retina virtual clinic utilising a diagnostic hub during COVID-19. 10.1038/s41433-021-01510-4 PMC8023775 33824508

[10] Chua PY, Sandinha MT, Steel DH. Idiopathic epiretinal membrane: progression and timing of surgery. Eye (Lond). 2022;36(3):495–503. 10.1038/s41433-021-01681-0. Epub 2021 Jul 21. PMID: 34290446; PMCID: PMC9074182. 34290446 10.1038/s41433-021-01681-0 PMC9074182

[11] Harby 11A, Ali L, Rajai Z, Roberts A, Peto SA, Leung T, Gray I, Hay J, Arora G, Keane AK, Cohen PA, Sagoo VML, Balaskas M. Prospective validation of a virtual clinic pathway in the management of choroidal Naevi: the NAEVUS study report 1: safety assessment. Br J Ophthalmol. 2022;106(1):128–34. 10.1136/bjophthalmol-2020-317371. Epub 2020 Oct 9. PMID: 33037007. 33037007 10.1136/bjophthalmol-2020-317371

[12] Clarke J, Puertas R, Kotecha A, Foster PJ, Barton K. Virtual clinics in glaucoma care: face-to-face versus remote decision-making. Br J Ophthalmol. 2017; 101(7):892–5. 10.1136/bjophthalmol-2016-308993 10.1136/bjophthalmol-2016-308993 27729310

[13] Halajyan CP, Thomas J, Xu B, Gluckstein J, Jiang X. Telemedicine [preprint]. [preprint].e care during [preprint].e COVID-19 [preprint].ndemic: A [preprint].view of [preprint].tient & [preprint].ysician [preprint].rspectives. MedRxiv [Preprint]. 2024 Oct 27:2024.10.25.24316160. doi: 10.1101/2024.10.25.24316160. PMID: 39502667; PMCID: PMC11537333.

[14] Saeed SA, Masters RM. Disparities in health care and the digital divide. Curr Psychiatry Rep. 2021;23(9):61. 10.1007/s11920-021-01274-4. PMID: 34297202; PMCID: PMC8300069. 34297202 10.1007/s11920-021-01274-4 PMC8300069

